# Lowering lifestyle‑related cancer risk through adherence to the 2018 WCRF/AICR recommendations: insights and implications

**DOI:** 10.1186/s44263-024-00037-6

**Published:** 2024-02-02

**Authors:** Xiaotao Zhang

**Affiliations:** 1 Institute for Translational Epidemiology, Icahn School of Medicine at Mount Sinai, New York, NY USA; 2 Division of Liver Diseases, Department of Medicine, Icahn School of Medicine at Mount Sinai, New York, NY USA; 3 Cancer Prevention and Control, The Tisch Cancer Institute, Icahn School of Medicine at Mount Sinai, New York, NY USA

A landmark UK BioBank study utilizing the WCRF/AICR scoring system has found a protective relationship between following these guidelines and the incidence of various lifestyle-related cancers. This comment delineates the UK BioBank study’s significant findings and also contextualizes them within the global effort to mitigate lifestyle-related cancer risks.

## Background

The global burden of cancer remains a paramount concern, with lifestyle-related cancers representing a significant proportion. Recognizing the critical role of modifiable factors in cancer prevention, the 2018 World Cancer Research Fund/American Institute for Cancer Research (WCRF/AICR) Cancer Prevention Recommendations were updated to incorporate the latest scientific evidence in the field [1]. In an era where lifestyle-related cancers are increasingly prevalent, understanding and implementing these recommendations is more critical than ever. These recommendations provide guidance on diet, physical activity, body weight management, and alcohol consumption with the aim of reducing cancer risk.

The relationship between cancer prevention and lifestyle has been extensively studied, with research consistently demonstrating that healthier behaviors can significantly lower the risk of developing certain types of cancer. The WCRF/AICR recommendations synthesize these findings into actionable advice. For example, a systematic review and meta-analysis and a UK biobank study highlighted the inverse association between higher adherence to these recommendations and the risk of overall cancer, breast, colorectal, kidney, pancreatic, uterine, esophageal, stomach, liver, and lung cancers, among others [2, 3].

## The UK Biobank study

A recent study published in BMC Medicine, using the UK Biobank cohort has provided additional evidence further supporting the protective effect of adherence to the WCRF/AICR guidelines [4]. This investigation was the first to apply the comprehensive scoring system of the 2018 recommendations to a UK cohort, examining its impact on all lifestyle-related cancers. Its findings revealed that an increased adherence score was associated with a lower risk of cancer, with hazard ratios indicating a 7–30% risk reduction for specific cancers, such as breast, colorectal, and gallbladder. This study underscores the potential of lifestyle changes as a crucial strategy in cancer prevention and represents a significant advancement over prior research by applying a comprehensive scoring system to a large and diverse cohort, offering more robust and generalizable findings.

The UK Biobank stands out as an exemplary resource in epidemiological research [5]. With over half a million participants and rich data collection on dietary intake, lifestyle factors, and biological samples, it provides a comprehensive picture of the interplay between lifestyle and cancer risk. Although self-reported dietary data may introduce recall bias and the data collection at a single time point might not reflect longitudinal dietary habits, the extensive scope and depth of the UK Biobank’s data are unparalleled. Findings based on these data are particularly influential, informing public health policies and cancer prevention strategies.

## Lifestyle-related cancers

Lifestyle-related cancers, which arise from modifiable behaviors, such as diet and exercise, represent a crucial target for prevention strategies. Addressing these behaviors can significantly diminish the risk of developing various cancers, making them key focal points in cancer prevention efforts. These modifiable behaviors not only affect individual health but also have substantial implications for healthcare systems and society, making preventive strategies a cornerstone of public health initiatives. The WCRF/AICR guidelines offer evidence-based recommendations for reducing the risk of these cancers. Preventative measures highlighted include smoking cessation, sun protection, maintaining a healthy weight, regular exercise, and a balanced diet rich in plant-based foods [1]. Public health initiatives that promote these recommendations can significantly impact cancer incidence and overall health.

## Evolution of the WCRF/AICR guidelines

The shift from the 2007 [6] to the 2018 WCRF/AICR guidelines [1] reflects the dynamic nature of nutrition and cancer prevention research. The latest guidelines offer more precise recommendations, with a notable emphasis on whole grains, vegetables, fruits, and limits on processed foods, red meats, and alcohol. They also advocate for breastfeeding, discourage supplement use and alcohol drinking, limit consumption of sugar-sweetened drinks, and suggest increased fiber intake (from 25 g daily intake recommendation before to 30 g daily intake recommendation currently). Furthermore, the guidance was refined by shifting from a general recommendation of ‘eat less salt’ to a more targeted and explicit emphasis on reducing salt intake, especially in processed foods (Table 1). This evolution signifies a move from a focus on individual nutrients to overall dietary patterns, aligning with contemporary research that underscores the synergy of dietary components. These evolving guidelines mirror the dynamic progression of nutritional science, offering refined strategies that are pivotal for effective cancer prevention in a changing world.

**Table 1:** Comparison of the 2007 and 2018 WICF/AICR guidelines

**Aspect**	**WICF/AICR 2007 guidelines**	**WICF/AICR 2018 guidelines**
**Physical activity**	Encouraged regular physical activity as a general goal	Specifies at least 150 min of moderate or 75 min of vigorous exercise per week
**Dietary guidelines**	Suggested a plant-based diet but was less specific about food types and amounts	More detailed on whole grains, vegetables, fruits, beans, and clear limits on fast foods, red and processed meats
**Alcohol consumption**	Recommended minimizing alcohol intake	For cancer prevention, it is best not to drink alcohol
**Dietary patterns vs. individual foods**	Focused on individual nutrients and foods	Emphasizes overall dietary patterns for combined effect on cancer risk
**Dairy consumption and cancer risk**	Less conclusive evidence on dairy consumption and its association with cancer	Includes updated evidence, with nuanced discussions on dairy consumption and cancer
**Breastfeeding**	Highlighted breastfeeding as a recommendation	Continues to emphasize breastfeeding for its protective effect against breast cancer
**Supplements**	Advised caution with dietary supplements	Advocates meeting nutritional needs through diet alone rather than supplements for cancer prevention
**Salt consumption**	Recommendation to eat less salt	Broadened and specified focus on reducing salt intake, particularly in processed foods
**Sugar-sweetened Drinks**	No specific mention of sugar-sweetened drinks	Added recommendation to limit consumption of sugar-sweetened drinks
**Dietary fiber intake**	A cut-off of consuming at least 25g per day of dietary fiber to meet the recommendation	Increased dietary fiber intake recommendation to at least 30g per day

## Comparison with other dietary indices/guidelines

In examining the role of diet in cancer prevention, the WCRF/AICR recommendations stand out for their specific focus on cancer risk reduction. Unlike the broader scope of the Healthy Eating Index-2020 [7], which evaluates diet quality based on the Dietary Guidelines for Americans (DGA), the WCRF/AICR recommendations are grounded in extensive research explicitly linking dietary patterns to cancer prevention. While the Dietary Inflammation Index also contributes valuable insights by measuring diet-induced inflammation, a known cancer risk factor, it does not exclusively focus on cancer prevention [8]. Similarly, the 2020 American Cancer Society Guidelines offer comprehensive advice on diet and physical activity for cancer prevention but include broader lifestyle considerations beyond dietary factors [9]. In contrast, the 2020–2025 DGA provide general health recommendations, lacking the cancer-specific emphasis inherent in the WCRF/AICR guidelines [10]. This distinctive focus of the WCRF/AICR on dietary elements directly associated with cancer risk, such as specific limits on red meat intake and avoidance of sugary drinks and alcohol, underscores their unique contribution to the field of cancer prevention research. Understanding the unique position of the WCRF/AICR guidelines among other dietary indices can aid healthcare professionals in formulating more targeted and effective dietary recommendations for cancer prevention.

## Future research directions

The established protective link between adherence to the WCRF/AICR recommendations and reduced lifestyle cancer risk marks the beginning of an explorative journey in cancer research. This new phase demands a deeper investigation into the molecular interactions influenced by lifestyle choices. Future studies should aim to unravel how lifestyle factors molecularly affect cancer risk, explore the complex relationship between dietary patterns and molecular factors, and examine cancer prevention effectiveness across various populations. This in-depth exploration is vital for comprehending the biological mechanisms that underlie these guidelines and for developing personalized cancer prevention strategies. A collaborative and dedicated research approach is needed to decode the intricate nexus of lifestyle factors, molecular biology, and cancer risk.

## Conclusions

Compelling evidence in favor of the 2018 WCRF/AICR Cancer Prevention Recommendations highlights their critical role in reducing the risk of various cancers. This is further reinforced by findings from this UK Biobank study [4], showcasing the transformative impact of lifestyle changes in cancer prevention. As we transition into the era of precision medicine, it becomes essential to thoroughly evaluate how current dietary guidelines align with various nutritional frameworks. A key aspect of this examination involves delving into the molecular basis of these guidelines, with the aim of customizing dietary recommendations to meet individual health needs, thereby enhancing cancer prevention strategies. Undertaking such research is crucial in broadening our understanding of cancer risks and developing more effective prevention methods. These insights will guide public health policies toward more effective strategies to address the worldwide cancer challenge. In an era increasingly burdened by cancer, the 2018 WCRF/AICR guidelines emerge as a vital guidepost. Harmonizing scientific research, public health measures, and individual behaviors with these recommendations is a promising path toward significantly reducing global cancer incidence.

## Data Availability

No datasets were generated or analysed during the current study.
